# The Green Lacewing, *Chrysoperla carnea*: Preference between Lettuce Aphids, *Nasonovia ribisnigri*, and Western Flower Thrips, *Frankliniella occidentalis*

**DOI:** 10.1673/031.013.9401

**Published:** 2013-09-30

**Authors:** Govinda Shrestha, Annie Enkegaard

**Affiliations:** Department of Agroecology, Science and Technology, Aarhus University, Research Centre Flakkebjerg, Forsøgsvej 1, DK-4200 Slagelse, Denmark

**Keywords:** Aphididae, biological control, Chrysopidae, prey ratios, Thripidae

## Abstract

This study investigated the prey preference of 3^rd^ instar green lacewing, *Chrysoperla carnea* Stephens (Neuroptera: Chrysopidae), between western flower thrips, *Frankliniella occidentalis* (Pergande) (Thysanoptera: Thripidae), and lettuce aphids, *Nasonovia ribisnigri* (Mosley) (Hemiptera: Aphididae) in laboratory experiments at 25 ± 1° C and 70 ± 5% RH with five prey ratios (10 aphids:80 thrips, 25 aphids:65 thrips, 45 aphids:45 thrips, 65 aphids:25 thrips, and 80 aphids:10 thrips). Third instar *C. carnea* larvae readily preyed upon both thrips and aphids, with thrips mortality varying between 40 and 90%, and aphid mortality between 52 and 98%. *Chrysoperla carnea* had a significant preference for *N. ribisnigri* at two ratios (10 aphids:80 thrips, 65 aphids:25 thrips), but no preference for either prey at the other ratios. There was no significant linear relationship between preference index and prey ratio, but a significant intercept of the linear regression indicated an overall preference of *C. carnea* for aphids with a value of 0.651 ± 0.054. The possible implications of these findings for control of *N. ribisnigri* and *F. occidentalis* by *C. carnea* are discussed.

## Introduction

The western flower thrips, *Frankliniella occidentalis* (Pergande) (Thysanoptera: Thripidae), and the lettuce aphid, *Nasonovia ribisnigri* (Mosley) (Hemiptera: Aphididae), are two economically important pests of lettuce (Blackman and Eastop 1984; Palumbo 1998; Natwick et al. 2007). Both pests are categorized as r-selected species with high reproductive capacity, parthenogenesis, and short generation time (Mound and Teulon 1995; Tatchell 2000; Diaz et al. 2010). *Frankliniella occidentalis* damages lettuce plants by scarring edible leaves and causing rib discoloration, while *N. ribisnigri* causes leaf distortion and reduces seedling vigor (Palumbo 1998). Their cryptic feeding and ability to act as major vectors for viral diseases are other negative attributes of these pests (Blackman and Eastop 1984; Yudin et al. 1987; Yudin et al. 1988). Finally, both pests are considered cosmetic pests (Palumbo 2000; Kift et al. 2004) because their presence in harvested products reduces the market value of the products.

The feeding preferences of *N. ribisnigri* for heart leaves and the cryptic behavior of *F. occidentalis* make them difficult to control with insecticides (Seaton et al. 1997; Parker et al. 2002; Stufkens and Teulon 2003). Using biological control would be an alternative control strategy for these pests. Several beneficial species have been studied for their potential to control either aphids or thrips, and many are commercially available and applied in practice (Powell and Pell 2007; Cloyd 2009). Among the more polyphagous species that might have a potential for contributing to control of both aphids and thrips is the green lacewing, *Chrysoperla carnea* Stephens (Neuroptera: Chrysopidae).

*Chrysoperla carnea* occurs naturally in a wide range of agroecosystems and is commercially available in Europe and North America (Wang and Nordlund 1994; Tauber et al. 2000). It has primarily been used through augmentative release to control various aphid species in greenhouses and outdoor crops (Scopes 1969; Tulisalo and Tuovinen 1975; Turquet et al. 2009). However, this species is a generalist predator, and is also known to predate on other soft-bodied arthropods, including scale insects, leafhoppers, whiteflies, psyllids, thrips, lepidopterans, and mites (Principi and Canard 1984). In field studies, satisfactory results were reported for *C. carnea* control of citrus thrips, *Scirtothrips citri* (Khan and Morse 1999a), leafhoppers, *Erythroneura variabillis* (Daane et al. 1996), and tobacco budworms, *Heliothis virescens* (Ridgway and Jones 1968).

The predation capacity of *C. carnea* towards the lettuce aphid has recently been examined (Shrestha 2011), but no information is available about the prey preferences of *C. carnea* towards *N. ribisnigri* and *F. occidentalis*, which appear simultaneously in lettuce fields. The prey preference of a predator directly affects the control efficiency of its various prey (Xu and Enkegaard 2009); thus, knowledge on preference is important to determine the potential of predators in situations in which several pest species are present in the crop of interest (Enkegaard et al. 2001). Consequently, the primary objective of this study was to evaluate the prey preference of *C. carnea* between *N. ribisnigri* and *F. occidentalis*.

## Materials and Methods

### Plants and insects

Iceberg lettuce, *Lactuca sativa* L. (Asterales: Asteraceae) var. ‘Mirette RZ’ was grown in plastic pots filled with a mix of perlite andvermiculite and maintained in a controlled environment glasshouse (15–20° C, 55–70% RH, 16:8 L:D) at Research Centre Flakkebjerg, Aarhus University, Denmark.

*Nasonovia ribisnigri* and *F. occidentalis* were reared separately on plants of lettuce and bean, *Phaseolus vulgaris* L. (Fabales: Fabaceae) var. “Montano”, respectively, in nylon net cages (68 × 75 × 82 cm) and maintained in a controlled environment glasshouse compartment (20 ± 1° C or 25 ± 1° C, respectively, 16:8 L:D, 55–70% RH). *Nasonovia ribisnigri* was originally supplied from Dr. Beatriz M. Diaz (Department of Plant Protection, CCMA-CSIC, Madrid, Spain). *Frankliniella occidentalis* had been reared at Research Centre Flakkebjerg, Aarhus University, Denmark, for 10 years.

One- to two-day-old 2^nd^ instar *C. carnea* larvae as well as eggs of the flour moth, *Ephestia kuehniella* Zeller (Lepidoptera: Pyralidae), were obtained from EWH Bio Production (www.bioproduction.dk). The *C. carnea* larvae were reared individually to the 3^rd^ instar on *E. kuehniella* eggs in Petri dishes (diameter: 5.5 cm) in a climate cabinet at 25 ± 1° C, 70 ± 5% RH, and 16:8 L:D. The larvae were transferred to new Petri dishes with an excess of *E. kuehniella* eggs at two-day intervals. The 3^rd^ instar larval stage was ascertained on the basis of morphology and developmental time (Scopes 1969; Butler and Ritchie 1970; Tauber 1974; Gepp 1984). One day before the experiment, 3^rd^ instar larvae were starved for 24 hr by keeping them individually in Petri dishes in a climate cabinet at the same conditions as above.

### Prey preference experiment

A circular lettuce leaf disc (diameter: 5 cm) was placed at the bottom of a Petri dish (8.5 cm diameter) lined with a thin layer of solidified agar solution (10%). The edge of the leaf disc was sealed with agar to prevent thrips larvae and aphid nymphs from hiding beneath the leaf. *N. ribisnigri* nymphs (3^rd^ and 4^th^ immature stages) and *F. occidentalis* larvae (1^st^ and 2^nd^ instars) were gently transferred with a fine camel hair brush from the rearings to the Petri dish. Aphid nymphs were introduced first and allowed to settle for 0.5–1 hr prior to introduction of thrips larvae to the dish. Subsequently, one starved *C. carnea* larva was placed in a Petri dish, which was then sealed with parafilm. The Petri dishes were placed in a plexiglass box (30.5 × 22.0 × 5.5 cm), the bottom of which was covered with a saturated salt water solution to maintain70 ± 5% RH. The experiment was carried out in a climate cabinet at 25 ± 1° C, 70 ± 5% RH, and 3 hr light conditions. After 3 hr, the numbers of live aphid nymphs and thrips larvae were counted under a stereomicroscope. Based on a preliminary experiment five prey ratios (10 aphids:80 thrips, 25 aphids:65 thrips, 45 aphids:45 thrips, 65 aphids:25 thrips and 80 aphids: 10 thrips) were selected, and each was tested in 10–15 replicates. Control treatments without *C. carnea* (8–10 replicates/ratio) were also carried out.

### Data analysis

After correction of the observed mortalities with the respective control mortality (Abbott 1925), Manly's preference index (Manly 1974) was calculated for each ratio of offered prey:

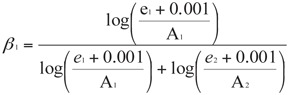
where *β_1_* is the preference to prey type 1, A_1_ and A_2_ are the number of offered prey type 1 and 2, and e1 and e2 are the numbers of prey type 1 and type 2 remaining after the experiment, respectively. The preference index (β) can attain values between 0 and 1. A β-value larger than 0.5 indicates a preference for prey type 1.

**Table 1.**
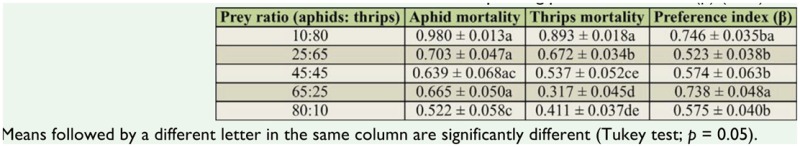
Mean mortality (± SE) inflicted by 3^rd^ instar larvae of *Chrysoperla carnea* on nymphs of *Nasonovia ribisnigri* and larvae of *Frankliniella occidentalis* when offered at different ratios, as well as the corresponding preferences indices (B) (± SE).

**Figure 1.**
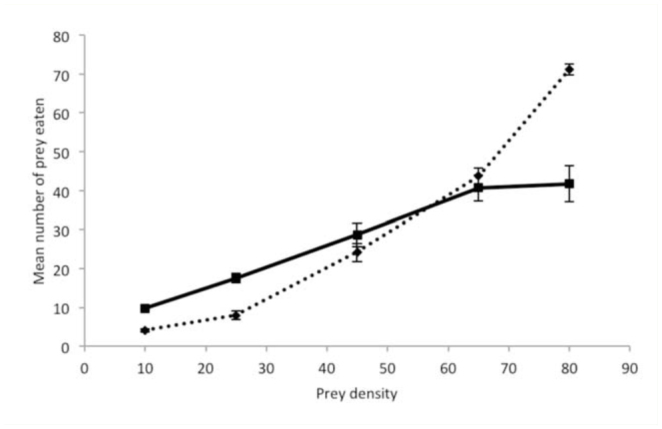
Mean predation rates (± SE) of *Chrysoperla carnea* in relation to the density of *Nasonovia ribisnigri* nymphs (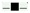) and *Frankliniella occidentalis* larvae (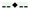) in relation to the density of either prey. High quality figures are available online.

Differences in mortality for each of the two prey species and differences in preference index between ratios were analyzed using a generalized linear mixed model (SAS/STAT, GLIMMIX procedure, SAS Institute Inc. 2004). The relationships between mortality and preferences indices, respectively, and the ratio of offered prey expressed as the proportion of aphids were analyzed with linear regression (SAS/STAT, REG procedure, SAS Institute Inc. 2004).

## Results

The predation rates of *C. carnea* on the two prey species is shown in Figure 1. The 3^rd^ instar larvae of *C. carnea* readily preyed upon both thrips and aphids, with thrips mortality varying between 40 and 90% and aphid mortality between 52 and 98% (Table 1). There were significant differences in thrips mortality (*F*_4, 46_ = 30.42, *p* < 0.0001) and aphid mortality (*F*_4, 45.4_ = 3.36, p = 0.0171) between the different ratios (Table 1). Both thrips and aphid mortalities decreased linearly as the proportion of aphids among the offered prey increased (thrips mortality: intercept 0.885 (± 0.047), *t* = 18.85, df = 64, *p* <0.0001, slope= 0.643, *t* = -8.38, df = 64, *p* < 0.0001; aphid mortality: intercept = 0.926 (± 0.058), *t* = 16.05, df = 64, *p <*0.0001, slope = -0.450 (± 0.094), *t* = -4.47, *p* <0.0007).

The preference index was significantly different from 0.5 for the ratios (aphids:thrips) 10:80 (*t* = 3,94, *p* = 0.0002, df = 60) and 65:25 (*t* = 4.75, *p* <0.0001, df = 60), whereas no significant differences were observed at the ratios 25:65 (*t* = 0,43, *p* = 0.6659, df = 60), 45:45 (*t* =1,55, *p* = 0.1263, df = 60), and 80:10 (*t* = 1.57, *p* = 0.1227, df = 60). There was no significant linear relationship between preference index and ratio (*F* = 0.17, df = 64, slope = - 0.037, *t* = - 0.41, *p* = 0.68), but the significant intercept (*t* = 12.04, *p* < 0.0001) of the linear regression indicated that the linear model predicts an overall preference of *C*.

**Figure 2.**
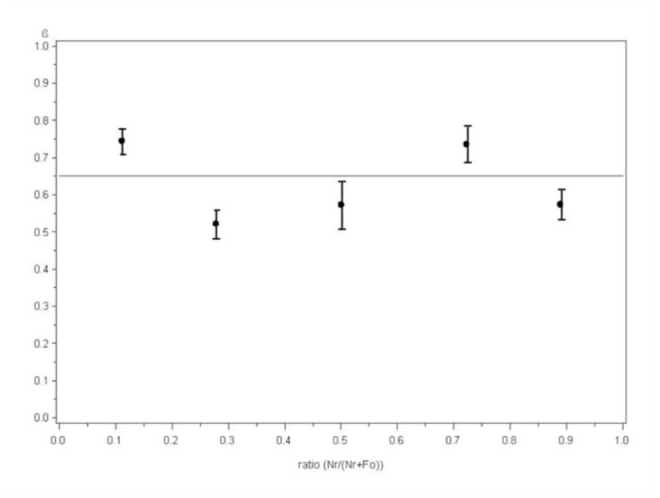
Mean prey preference index (± SE) of *Chrysoperla carnea* for *Nasonovia ribisnigri* when provided with varying ratios (10:80, 25:65, 45:45, 65:25, and 80:10) of *N. ribisnigri* nymphs and *Frankliniella occidentalis* larvae. High quality figures are available online. *carnea* for aphids at a value of 0.651 ± 0.054 (Figure 2).

## Discussion

### Predation on aphids and thrips

The results have shown that *C. carnea* readily prey upon both *N. ribisnigri* and *F. occidentalis*, with a maximum predation rate at the ratio with the highest number of each of the two prey species, respectively, being approximately 42 aphids and 71 thrips during the 3 hr experimental period.

Since two prey species were provided simultaneously, direct comparisons with other studies on predation by *C. carnea* in single-prey experiments offering aphids or thrips are not strictly valid, but may give an indication of the comparable predation capacity of *C. carnea*. Thus, the predation of *N. ribisnigri* by *C. carnea* observed in this study at the aphid:thrips ratio of 45:45 is in accordance with the study on functional response of *C. carnea* towards *N. ribisnigri* conducted at 25° C (Shrestha 2011), in which a predation of 24 *N. ribisnigri* in 3 hr was observed at the highest provided density of 45 aphids. Although the other aphid densities provided in the functional response experiment (Shrestha 2011) are not directly comparable with those provided here, the predation of 10 and 17 *N. ribisnigri* at the aphids:thrips ratio of 10:80 and 25:65, respectively, found in this study, seems comparable with the predation of 9 and 13 *N. ribisnigri* at the density of 12 and 20 *N. ribisnigri*, respectively, in the functional response experiment. Thus, the predation capacity of *C. carnea* towards *N. ribisnigri* does not seem to be affected by the presence of *F. occidentalis* larvae.

The highest predation of *N. ribisnigri* observed in this study (approximately 43 attained at the aphids:thrips ratio of 80:10) is equivalent to about 230 *N. ribisnigri* per day, assuming 16 hr light and predation only during the light phase. This seems higher than that observed for predation by *C. carnea* on other aphid species, although comparisons are difficult due to differences between studies, especially in the experimental period but also in the number of prey offered and experimental conditions. Daily predation rates of 160 mealy plum aphids, *Hyalopterus pruni* (Atlihan et al. 2004), 138 cabbage aphids, *Brevicoryne brassicae* (Huang and Enkegaard 2010), and 110 green peach aphids, *Myzus persicae* (Scopes 1969), have been reported for 3^rd^ instar *C. carnea* at experimental conditions similar to the ones described here. However, it must be kept in mind that the predation during the first few hours after a starvation period is likely to be higher than that attained during the remaining feeding period (Skirvin and Fenlon 2003), i.e., the estimated daily predation by *C. carnea* on *N. ribisnigri* of 230 may be an overestimation.

The highest predation of *F. occidentalis* observed in this study was approximately 71, attained at the aphids:thrips ratio of 10:80, which is equivalent to about 380 *F. occidentalis* per day, again assuming 16 hr light and predation only during the light phase. This is in accordance with results from a study carried out by Khan and Morse (1999b) on predation of 3^rd^ instars of the closely related lacewing *Chrysoperla rufilabris* on citrus thrips larvae, *Scirtothrips citri*, of which 85 were consumed in 3 hr. No studies seem to have investigated the predation of 3^rd^ instar *C. carnea* on *F. occidentalis*. However, Bennison et al. (1998) reported a predation of 36 *F. occidentalis* larvae per day by 2^nd^ instar *C. carnea*. If Bennison et al. (1998) had studied the predation of 3^rd^ instar lacewings, a consumption predation of about 145–180 *F. occidentalis* per day could have been expected based on the much less voracious nature of 2^nd^ instars compared to 3^rd^ instars, which have been reported to prey on 4–5 times the amount of aphids or caterpillars consumed by 2^nd^ instars (Klingen et al. 1996; Huang and Enkegaard 2010). Our estimated daily consumption of 380 *F. occidentalis* seems to be much higher than this value. As mentioned above, one possible explanation is that the daily consumption from the present results may be overestimated due to a decline in pre dation rate with reduced hunger level. However, other factors, such as temperature, may also play a role.

### Prey preference

Third instar larvae of *C. carnea* only exhibited a significant preference of *N. ribisnigri* over *F. occidentalis* in two prey ratios (aphids:thrips), 10:80 and 65:25, while no clear indication of preference was found for the other ratios. There are no obvious explanations for these observations. However, taken across all ratios, *C. carnea* showed an overall preference for *N. ribisnigri* nymphs over *F. occidentalis* larvae that remained unchanged across all ratios, demonstrating that *C. carnea* did not switch preference in response to changing ratios of the prey species, a behavior often exhibited by polyphagous predators (Murdoch 1969; Albajes and Alomar 1999; Nachappa et al. 2006). However, an actual switching behavior may have been masked if starved *C. carnea* in the first part of the experimental period fed indiscriminately to satisfy their immediate hunger and reached a certain satiation level after which the remaining pre dation proceeded according to a preference for aphids (Sukhanov and Omelko 2002).

A preference for aphids over thrips is in accordance with earlier findings mentioned by Tulisalo (1984), citing a Russian paper by Advashkevich et al. (1972), although the actual aphid and thrips species are not specified. It can be speculated that *C. carnea* might consider *N. ribisnigri* nymphs as a higher-quality food, or that this prey is preferred due to its rather immobile nature and larger size and thus easier detectability. It may also be speculated that *C. carnea* reacts to chemical cues produced by *N. ribisnigri* (Pickett and Glinwood 2007).

### Conclusion

*Nasonovia ribisnigri* and *F. occidentalis* are two important pests in lettuce. Both pests may occur simultaneously in the same field and on the same leaves. The present results indicate that *C. carnea* has a potential for biological control of *N. ribisnigri* and may also contribute to control of *F. occidentalis*. This information is valuable in connection with augmentative biocontrol in lettuce fields and in glasshouse-grown lettuce. In addition, it may be of use in connection with development of conservation biocontrol strategies for control of *N. ribisnigri* in field-grown lettuce when this is combined with knowledge on the degree of *C. carnea* population enhancement through various conservation tactics. However, caution must be taken when predicting the performance of a predator under field conditions from small-scale laboratory experiments. Consequently, a full evaluation of the biocontrol potential of *C. carnea* against thrips and aphids in lettuce requires further investigations in which effects of, for example, plant architecture in relation to the cryptic behaviour of the two pest species, prey spatial distribution, and presence of other pest and beneficial species on the prey preference characteristics of *C. carnea* are examined.
